# Engineered Liposomal Delivery of *Human ACE2* Across the Blood–Brain Barrier Attenuated Neurogenic Hypertension

**DOI:** 10.3390/pharmaceutics17101329

**Published:** 2025-10-14

**Authors:** Yue Shen, Richard Nii Lante Lamptey, Gowthami Reddy Mareddy, Bivek Chaulagain, Jagdish Singh, Chengwen Sun

**Affiliations:** Department of Pharmaceutical Sciences, North Dakota State University, Fargo, ND 58105, USA; yue.shen.1@ndsu.edu (Y.S.);

**Keywords:** neurogenic hypertension, brain-targeted delivery, functionalized liposomes, angiotensin-converting enzyme 2, blood–brain barrier

## Abstract

The blood–brain barrier (BBB) restricts the entry of therapeutic agents into the brain cardiovascular regulatory region, potentially contributing to drug-resistant hypertension. **Objective**: The objective of this study was to overcome this limitation by modifying PEGylated liposomes with transferrin (Tf) to facilitate Tf receptor binding at the BBB and penetratin (Pen), a cell-penetrating peptide, to enhance neuronal uptake. **Methods**: This study evaluated the efficacy of Tf-Pen-liposomes in delivering angiotensin-converting enzyme 2 (ACE2) or EGFP (control) genes across the BBB in rats. In addition, the therapeutic effect of intravenous administration of Tf-Pen-Lip carrying plasmid DNA encoding ACE2 (Tf-Pen-Lip-pACE2) was tested in a neurogenic hypertension model induced by intracerebroventricular (ICV) infusion of angiotensin II (Ang II) via osmotic pump implantation and brain cannulation. **Results**: Conjugation with Tf and Pen significantly enhanced liposome-mediated gene transfection in cultured cells and increased transport across an in vitro BBB model. In vivo, intravenous administration of Tf-Pen-Lip-pACE2 or Tf-Pen-Lip-pGFP successfully elevated ACE2 or EGFP expression, respectively, in the hypothalamic paraventricular nucleus (PVN). Chronic ICV infusion of Ang II produced a sustained increase in blood pressure and heart rate, accompanied by sympathetic overactivation and elevated arginine vasopressin (AVP) secretion, hallmarks of neurogenic hypertension. Notably, intravenous Tf-Pen-Lip-pACE2 treatment dramatically attenuated Ang II–induced neurogenic hypertension, whereas Tf-Pen-Lip-pGFP had no effect on pressor responses, sympathetic activity, or AVP secretion. **Conclusions**: This dual-functionalized liposomal delivery system effectively transported the ACE2 gene across the BBB into the brain, increased ACE2 expression, and markedly attenuated neurogenic hypertension following systemic administration.

## 1. Introduction

Hypertension is the most significant prognostic risk factor for cardiovascular and renal morbidity and mortality. Approximately one billion people worldwide have hypertension, and nearly half of American adults (47.7%) have hypertension [1,2]. Despite aggressive campaigns promoting lifestyle changes and advances in drug therapy, hypertension remains a major health, emotional, and economic burden. This is partly because about 15% of hypertension patients are resistant to current antihypertensive drugs [3]. Therefore, there is an urgent need to develop novel therapeutic strategies to control drug-resistant hypertension.

Recent clinical studies have demonstrated that two surgical methods are effective for patients with resistant hypertension: renal sympathetic denervation, which reduces sympathetic innervation to the kidney [4], and pacemaker implantation, which stimulates baroreceptors to reduce sympathetic nerve activity via baroreflex activation [5]. However, these surgical interventions are associated with irreversible complications that may exceed patients’ tolerance levels [6,7]. Nevertheless, those clinical trials on these surgical methods have provided valuable insights, demonstrating that targeting central nervous system (CNS)-mediated sympathetic overactivation is an effective strategy for treating resistant hypertension. The sympathetic nervous system is regulated by key brain cardiovascular regulatory regions, such as the paraventricular nucleus (PVN) in the hypothalamus and the nucleus tractus solitarii (NTS) in the brainstem [8,9]. Consequently, it is essential to develop a novel therapeutic approach that targets neuronal modulators in the CNS to reduce sympathetic nervous activity for the effective treatment of resistant hypertension.

The brain renin-angiotensin system (RAS) plays a crucial role in regulation of blood pressure (BP) and development of hypertension. A recently identified member of the RAS, angiotensin-converting enzyme 2 (ACE2), is present in the brain cardiovascular regulatory regions, where it converts angiotensin II (Ang II) to Ang-(1-7), one of the mechanisms contributing to ACE2’s antihypertensive effects [10,11]. ACE2 is central to the antihypertensive axis of the RAS, a concept supported by substantial evidence. Brain-selective overexpression of ACE2 has been shown to attenuate hypertension development and improve autonomic function through transgenic approaches [12]. Additionally, our previous study demonstrated that viral vector-mediated overexpression of ACE2 via direct brain injection into the brain cardiovascular regulatory regions significantly attenuates hypertension and elevated sympathetic nerve activity in hypertensive animal models [13]. However, direct brain injection techniques used in animals cannot be applied to humans due to the risk of brain damage and infection. Furthermore, a drug’s accessibility to the brain is hindered by the presence of the blood–brain barrier (BBB). Thus, an essential step in developing effective treatments is the creation of a carrier system capable of efficiently delivering therapeutic agents across the BBB to enhance ACE2 expression in the brain.

The BBB serves as a critical barrier that restricts the transport of molecules, including therapeutic agents, into the brain. In the present study, we developed a liposome-based delivery system to transport the ACE2 gene across the BBB and enhance ACE2 expression in the brain. To improve liposome-mediated brain-targeted delivery efficacy, the liposome surface was modified with transferrin (Tf) and penetratin (Pen). Tf is a serum glycoprotein that facilitates iron transport into the brain via Tf-receptor-mediated transcytosis [14,15]. Tf receptors are abundant in the brain capillary endothelial cells but scarce in other vascular tissues, making Tf modification a promising strategy for brain-targeted delivery [16]. Pen, a 16-mer peptide derived from Drosophila Antennapedia, enhances gene delivery efficiency across biological membranes and protects against endosome-mediated degradation [17,18]. Therefore, the present study aimed to characterize the Tf-Pen-liposome-mediated gene delivery system, evaluate the transport efficacy of this system across the BBB, and determine the effects of centrally delivering the ACE2 gene across the BBB using Tf-Pen-liposomes on neurogenic hypertension.

## 2. Materials and Methods

### 2.1. Animals and Materials

Sprague-Dawley (SD) rats (250–320 g, either sex) used in this study were purchased from Charles River Laboratories and housed under controlled conditions with a 12-h light/dark cycle. Food and water were provided ad libitum. One-day-old SD rats used for neuronal and glial cell cultures were obtained from our breeding colony. All experimental protocols were approved by the North Dakota State University Institutional Animal Care and Use Committee (IACUC) and conducted in accordance with the Declaration of Helsinki and the Guide for the Care and Use of Laboratory Animals.

Fetal bovine serum (FBS), B27, Lissamine rhodamine, and GlutaMax were purchased from Thermo Fisher Scientific. DSPE-PEG2000-NHS was obtained from Biochempeg Scientific Inc. Dulbecco’s Modified Eagle Medium (DMEM) was purchased from Fisher Scientific. Chitosan (30 kDa) was obtained from Glentham Life Sciences (Corsham, UK). The Pen peptide was purchased from Zhejiang Ontores Biotechnologies Co., Ltd. (Zhejiang, China). Lentiviral vector plasmids containing EGFP (pGFP) cDNA or human ACE2 (pACE2) cDNA were purchased from Vector Builder (Chicago, IL, USA). The GenCatch™ Plasmid DNA Mini-Prep Kit was obtained from Epoch Life Science (Fort Bend County, Texas, USA). Alanine aminotransferase (ALT), aspartate aminotransferase (AST), and creatinine assay kits were purchased from Abcam (Waltham, MA, USA). Poly-L-lysine, cytosine arabinoside, EDTA, HEPES, Triton X-100, cholesterol, Hoechst 33342, DNase I, Holo-transferrin, and other reagents were purchased from Sigma-Aldrich (Burlington, MA, USA).

### 2.2. Preparation of Cell Cultures and Plasmids

Primary neuronal cultures were prepared from the hypothalamus of neonatal rat pups as previously described [19]. Briefly, hypothalamic cells were dissociated using trypsin and DNase I, then resuspended in Neurobasal medium supplemented with 5% fetal bovine serum (FBS), B27 (2 μL/mL), and GlutaMax, and maintained at 37 °C. Following cell quantification, the suspension was plated onto poly-l-lysine–coated dishes and cultured in a humidified incubator (37 °C, 5% CO_2_). Cytosine arabinoside (5 µM) was added for two days to inhibit glial cell proliferation. Primary astrocytes and pericytes were isolated from the hypothalamus of neonatal rat pups as described previously [20,21] and maintained in DMEM supplemented with 10% FBS. The endothelial cell line bEnd.3 was purchased from ATCC and cultured in DMEM containing 10% FBS and 100 U/mL penicillin–streptomycin. Lentiviral vector plasmids containing EGFP (pGFP) cDNA or human ACE2 (pACE2) cDNA in *E. coli* were amplified and isolated using the GenCatch™ Plasmid DNA Mini-Prep Kit (Epoch Life Science, Sugar Land, Texas, USA) according to the manufacturer’s instructions.

### 2.3. Preparation of Liposomes

Functionalized liposomes were prepared as previously described [22]. Briefly, holo-transferrin (Tf) was coupled to terminal NHS-activated DSPE-PEG2000 phospholipid (125 μg Tf/μmol phospholipid) via nucleophilic substitution in anhydrous dimethylformamide (DMF). The reaction mixture was adjusted to pH 8.0–8.5 with triethylamine and stirred at room temperature for 24 h to form Tf–DSPE-PEG micelles. Unbound Tf was removed by filtration through a Sephadex G-100 column. Similarly, Pen and DSPE-PEG2000-NHS were dissolved in DMF to form Pen–DSPE-PEG. The reaction mixture was adjusted to pH 8.0–8.5 with triethylamine and stirred at room temperature for 3 days. Unconjugated Pen was removed by dialysis against deionized water for 48 h.

Prepared Pen–DSPE-PEG (4 mol%), DOPE (45 mol%), DOTAP (45 mol%), and cholesterol (2 mol%) were dissolved in chloroform/methanol (2:1, *v*/*v*) and evaporated to form a thin lipid film, which was then rehydrated in HEPES buffer. Liposomes were formed by sonication of the buffer. Pen-liposomes and Tf–DSPE-PEG micelles were mixed and stirred overnight to yield Tf–Pen–liposomes. The liposomes were passed through a Sephadex G-100 column to remove free Tf and further purified using a 0.2 μm polycarbonate membrane. To enhance plasmid stability prior to encapsulation, we prepared a chitosan–pDNA complex by dissolving 1% chitosan (*w*/*v*, MW 30 kDa, deacetylation degree 85–90%) in 0.2 M acetate buffer (pH 4.5), followed by the addition of pDNA at an N/P ratio of 5:1. This chitosan–pDNA complex was then encapsulated into liposomes using the “post-insertion” method, which provides high encapsulation efficiency and ensures liposomal stability.

### 2.4. Characterization of Liposomes

The hydrodynamic size, polydispersity index (PDI), and zeta potential of the synthesized liposomes were measured by dynamic light scattering using a Zetasizer Nano ZS (Malvern Instruments, Malvern, UK) equipped with a 5 mW He–Ne laser (633 nm) at 25 °C. Data were collected at a scattering angle of 90°. Encapsulation efficiency (EE) of pACE2- or pGFP-loaded liposomes was determined using the DNA-intercalating dye Hoechst 33342 (0.15 μg/mL). Briefly, 0.5% (*v*/*v*) Triton X-100 was added to disrupt liposome membranes and release the total amount of encapsulated pACE2. Free, unencapsulated pACE2 or pGFP was measured in samples without Triton X-100. Fluorescence intensity was recorded at 354/458 nm (excitation/emission). EE% was calculated using the following formula: EE% = (A − B)/A × 100, where A is the fluorescence intensity of total pDNA and B is the fluorescence intensity of unencapsulated pDNA.

To evaluate liposomal protection of encapsulated pDNA against nuclease degradation, we performed a DNase protection assay. Liposomes (plain, Pen, Tf, and Tf–Pen) containing 1 μg pGFP were incubated with 1 U DNase I in PBS at 37 °C for 60 min. Naked pDNA (pGFP, 1 μg) served as a positive control under the same conditions. DNase activity was terminated by adding EDTA (100 mM, 5 μL). Encapsulated pDNA was released by adding heparin (5 mg/mL) and incubating for 2 h. The integrity of the released DNA was assessed by electrophoresis on a 0.8% (*w*/*v*) agarose gel.

Gene-transfer efficiency of liposomes was evaluated in bEnd.3 cells, primary neuronal cultures, and primary astrocyte cultures after treatment with Lip–pGFP, Pen–Lip–pGFP, Tf–Lip–pGFP, or Tf–Pen–Lip–pGFP (100 nM). Each liposomal formulation containing 1 μg chitosan–pGFP complex was added to cells in serum-free medium and incubated for 4 h. The medium was then replaced with complete serum-containing medium and cells were further incubated for 48 h. EGFP protein expression was examined by fluorescence microscopy, and fluorescence intensity was quantified using ImageJ. Results were normalized to the imaged area.

### 2.5. Preparation and Evaluation of In Vitro BBB Model

To evaluate the transport efficiency of liposomes across the barrier layers, the in vitro BBB model was constructed using a combination of bEND.3 (endothelial cell), primary astrocyte, pericyte, and neuronal cultures (EAPN). Astrocytes and pericytes were seeded (1.5 × 10^4^ cells/cm^2^) on the bottom side of a collagen-coated polyethylene terephthalate (PET) membrane of trans-well inserts (BD Biosciences, Milpitas CA, USA) in DMEM with 10% FBS. The cells were allowed to adhere firmly to the membrane overnight. Thereafter, the endothelial cells were seeded on the inside of the culture inserts that were placed in 35-mm culture dishes and cultured in culture medium for 3 days, allowing the formation of a tight barrier layer. The primary neurons were cultured in 35-mm culture dishes as described above. After 3-day culture, the transwell inserts with co-cultures were transferred to neuronal culture dishes allowing neurons to contact and interact with astrocytes and pericytes and incubated in culture medium for an additional 5 days to further increase the integrity of the barrier layer.

The barrier layer’s integrity was evaluated by measuring the TEER (trans-endothelial electrical resistance) using the patch-clamp technique as described in our previous publication [23]. The TEER was calculated using the equation:

TEER = (R_total_ − R_blank_) × A_membrane_

In the equation, R_total_ is the total resistance measured across the cell layers; R_blank_ is the resistance of a cell-free insert; and A_membrane_ is the surface area of the transwell membrane (cm^2^).

In addition, the barrier layers’ permeability across the BBB model was evaluated by measuring the flux of sodium fluorescein (Na-F) across the barrier layer; and calculated using the equation:

1/Pe = 1/Pt − 1/Pf (cm/s)

In the equation, Pt is the permeability coefficient of the total system and Pf for the permeability coefficient across the cell-free inserts. Permeability coefficient for each liposomal formulation dividing the amount of liposomes transported per min (µg/min) through total system (Pt) or cell-free insert (Pf) by the surface area of the trans-well membrane (cm^2^).

### 2.6. Liposome-Mediated Delivery Across the BBB: In Vitro and In Vivo Evaluation

The transport efficacy of liposomes (plain, Pen, Tf, and Tf-Pen) across in vitro BBB was evaluated in this co-cultured BBB model using lissamine rhodamine loaded in the liposomes as detailed in our previous publication [24]. Briefly, rhodamine-loaded liposomes were added to the upper chamber and incubated for 0.5, 1, 2, 4, 8, and 12 h in the incubator, respectively. The concentrations of the liposomes in the upper and lower compartments were detected by measuring the fluorescence intensity of the rhodamine using a spectrofluorometer (excitation wavelength: 560 nm, emission wavelength: 570 nm), and expressed as percent transport of liposomes.

The in vivo transport efficacy of liposomes across the BBB was evaluated by Tf-Pen-Liposomes loaded with pGFP in rats. The Tf-Pen-Liposomes loaded with pGFP (TP-pGFP, 500 μg/kg) or the liposomes only (control) were injected intravenously. The EGFP expression in the neurons within the brain of rats was detected using immunohistology approaches as described in our previous publication [25]. Briefly, the brains were collected at one week after injection and sectioned to brain slides using Cryostat (Leica Biosystems, Dear Park, IL, USA). The brain sections containing the PVN were stained with primary antibodies against NeuN (neuronal marker, Sigma MAB377, dilution 1:500) and EGFP (Thermo Fisher Scientific OSE00003G; dilution 1:500) overnight at 4 °C, followed by incubation with secondary antibodies (Thermo Fisher Scientific, Denver, CO, USA): Goat anti-rabbit IgG antibody Alexa Fluor 594 and Goat anti-mouse Alexa Fluor 488 for 2 h at room temperature. The brain sections were then washed with PBS/Tween and mounted with anti-bleaching medium on a glass coverslip. The fluorescence staining was detected using a confocal fluorescence microscope (Leica, DMi8, Dear Park, IL, USA) and analyzed using an ImageJ (version 1.54p) computer software.

### 2.7. Blood Pressure Measurement and Treatment Protocol of Rats

Blood pressure (BP) and heart rate (HR) were recorded using a noninvasive tail-cuff system (plethysmography) before and after treatments. Rats underwent at least two weeks of pre-training, during which they were placed in appropriately sized restrainers for 10 min daily to acclimate to the measurement environment and procedure, thereby minimizing stress. During recordings, body temperature was maintained at 32–35 °C using a heat lamp and warming pad, and monitored with an infrared thermometer. The rat tail was threaded through the occlusion cuff, positioned near the base of the tail without pressure, and the VPR cuff was placed within 2 mm of the occlusion cuff. BP and HR were measured daily using the CODA system (Kent Scientific, Torrington, CT, USA).

A neurogenic hypertension animal model was generated by chronic intracerebroventricular (ICV) infusion of Ang II for 4 weeks using the ALZET brain infusion kit and osmotic pumps (Alzet Osmotic Pumps, Campbell, CA, USA), as described previously [26]. Artificial cerebrospinal fluid (aCSF) served as the control and contained (in mM): NaCl 125, NaHCO_3_ 25, glucose 10, KCl 2.5, NaH_2_PO_4_ 1.2, CaCl_2_ 2, MgCl_2_ 1.2 (pH 7.4). All implantation procedures were performed under aseptic conditions. Rats were anesthetized with isoflurane and secured in a stereotaxic apparatus (David Kopf Instruments, Tujunga, CA, USA). After shaving and cleaning the skull with 70% ethanol and povidone-iodine, a burr hole was drilled at the coordinates of the left lateral ventricle (1.1 mm caudal to bregma, 1.5 mm lateral to midline, 4 mm below the skull surface). A 28-gauge infusion cannula was inserted into the ventricle, secured to the skull with tissue glue, and connected to a prefilled osmotic minipump implanted subcutaneously on the back. The incision was sutured and treated with antibiotic ointment. Rats were then removed from the stereotaxic frame and placed in prewarmed cages for recovery. Five days after initiating ICV infusion of Ang II (20 ng/min) or aCSF, two additional groups of Ang II-treated rats received intravenous injections of Tf–Pen–liposomes loaded with either pACE2 or pGFP (500 μg/kg) via the tail vein. Following injection, BP, HR, and water intake were recorded daily for an additional 23 days.

At the end of the 4-week treatment protocol, autonomic function was assessed in all four groups by measuring ΔHR and ΔMAP before and after intraperitoneal injection of atropine (muscarinic receptor antagonist, 1 mg/kg), propranolol (β-adrenoceptor blocker, 4 mg/kg), or chlorisondamine (ganglionic blocker, 5 mg/kg). Cardiovascular parameters were measured 3 h after drug administration to allow recovery. At the end of the experiment, brain tissue and blood were collected for ACE2 gene expression analysis and plasma hormone assays respectively.

### 2.8. ACE2 Gene Expression and Activity Measurements

ACE2 protein levels in the PVN were measured by Western blotting as previously described [26]. Briefly, PVN tissue was micro-punched from brain sections of rats after 4 weeks of treatment with control, Ang II, Ang II + Tf–Pen–Lip–pGFP, or Ang II + Tf–Pen–Lip–pACE2. Tissue samples were lysed in a lysis buffer, homogenized for 15 s, boiled for 3 min, ultrasonicated, and centrifuged. Supernatants were stored at −80 °C. Protein concentrations were determined using a protein assay kit (Bio-Rad Laboratories, Hercules, CA, USA). Equal amounts of protein (10 μg) were loaded onto 10% SDS-PAGE gels, separated, and transferred to nitrocellulose membranes at 100 V for 2 h. Membranes were washed in PBS-T, blocked in PBS-T containing 10% milk and 1% BSA for 2 h, and incubated overnight at 4 °C with anti-ACE2 primary antibody (Abcam, ab15348, 1:500). After washes, membranes were incubated with goat anti-rabbit IgG secondary antibody (Bio-Rad, 1706515, 1:5000) for 2 h at room temperature. Immunoreactive bands were visualized using enhanced chemiluminescence (ECL Western blotting detection kit, Amersham Pharmacia Biotech, Piscataway, NJ, USA) and quantified with ImageJ.

ACE2 mRNA expression in PVN tissue was determined by real-time PCR as described previously [27]. Total RNA was isolated from micro-punched PVN tissue using the RNeasy Mini Kit (Qiagen, Valencia, CA, USA). RNA purity and concentration were determined spectrophotometrically. For each reaction, 2 μg RNA was reverse-transcribed to cDNA (Qiagen), with genomic DNA removed by DNase I digestion. Real-time PCR was performed in 96-well plates with TaqMan PCR Master Mix (Applied Biosystems, Foster City, CA, USA). ACE2-specific probes were purchased from Applied Biosystems. Data were normalized to 18S rRNA expression.

ACE2 enzymatic activity in PVN tissue was measured using the quenched fluorescent substrate Mca-Ala-Pro-Lys(Dnp)-OH (AnaSpec, 60757, Fremont, CA, USA), as previously described [28]. Fluorescence was measured at 320 nm excitation and 405 nm emission using a microplate reader. Enzyme activity was normalized to protein concentration (μg) in each sample.

### 2.9. Evaluation of Histological and Physiological Effect of Tf-Pen-Lip-pACE2

To evaluate potential side effects of the liposomal formulation, rats were treated with either saline (control) or Tf–Pen–Lip–pACE2 (500 μg/kg, IV). After 4 weeks of treatment, rats were euthanized, and major organs (brain, heart, kidney, liver, lung, and spleen) as well as plasma were collected. Organs were sectioned using a cryostat (Leica Biosystems. Foster City, CA USA) and stained with hematoxylin and eosin (H&E). Histological images were examined with a reverse microscope (Olympus, San Jose, CA, USA) equipped with Infinity Capture and Analyze software.

Plasma biochemical markers were also assessed to determine systemic toxicity. Liver function was evaluated by measuring plasma alanine aminotransferase (ALT) and aspartate aminotransferase (AST) levels, and kidney function was assessed by measuring plasma creatinine. All assays were performed using ELISA kits (Abcam, Cambridge, MA, USA) according to the manufacturer’s instructions.

### 2.10. Statistical Analyses

Statistical analyses were performed using GraphPad Prism (version 10.1.2) Data are expressed as mean ± SEM. Comparisons between two groups were evaluated using a two-tailed Student’s *t*-test, while multiple group comparisons were analyzed by one-way or two-way ANOVA, followed by Newman–Keuls or Bonferroni post hoc tests as appropriate. Differences were considered statistically significant at *p* < 0.05. Exact *p* values are provided in the figure legends.

## 3. Results

### 3.1. Synthesis and Characterization of pDNA-Loaded Liposomes

The size, surface features, and shape of liposomes are critical for their biodistribution in vivo. It has been reported that nanoparticles with diameters ranging from 30 to 200 nm are commonly used for brain-targeted in vivo delivery, as this size range allows particles to remain in circulation while avoiding rapid renal clearance [29,30]. To further enhance BBB penetration and improve brain targeting, we modified the surface of liposomes with the cell-penetrating peptide Penetratin (Pen), Transferrin (Tf), or both, as described in the Methods.

**Figure 1:**
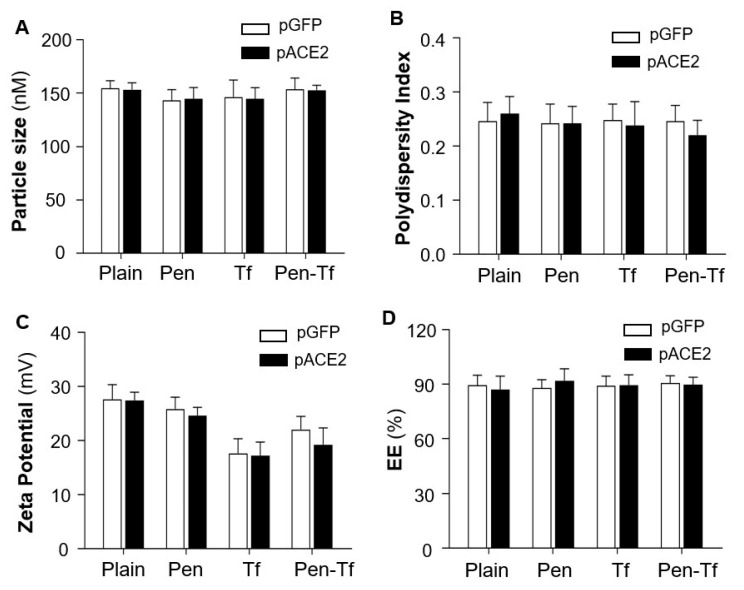
Characterization of liposomes loaded with pGFP or pACE2. (**A**). Hydrodynamic diameter of liposomes with surface modifications (Pen, Tf, or Pen + Tf) compared with unmodified (Plain) liposomes. (**B**). Polydispersity index of different liposomes. (**C**). Surface charge (zeta potential) of different liposomes. (**D**). Encapsulation efficiency (EE) of pDNA in liposomes, measured using Hoechst 33342 DNA staining. Data are presented as mean ± SEM (*n* = 5 independent preparations). No significant differences were observed between pGFP- and pACE2-loaded liposomes (*p* > 0.05).

The physicochemical properties of the liposomes were characterized by light scattering using a Zetasizer Nano ZS (Malvern Instruments, Malvern, UK). Results showed that the sizes of plain liposomes ranged from 140 to 160 nm. Surface modification with Tf, Pen, or both did not significantly alter particle size, which remained within the desirable range for brain-targeted delivery (Figure 1A). All liposome formulations had a polydispersity index (PDI) of less than 0.3, indicating a uniform particle distribution (Figure 1B).

Zeta potential measurements describe the net surface charge of liposome particles and can help predict in vivo interactions and formulation stability. Generally, liposomes with low absolute zeta potentials tend to aggregate more quickly and exhibit lower stability compared with those having higher zeta potentials [31]. The plain liposomes exhibited a positive zeta potential due to the incorporation of the cationic phospholipid DOTAP. Conjugation with positively charged Pen, negatively charged Tf, or both did not significantly alter the surface charge (Figure 1C).

Notably, efficient encapsulation of pACE2 or pGFP into liposomes was achieved for all formulations, with encapsulation efficiencies exceeding 85% (Figure 1D). These results confirm successful liposome functionalization, which is critical for achieving brain-targeted delivery across the BBB in the current study. Both pACE2- and pGFP-loaded liposomes were prepared in HEPES buffer at pH 7.4 and used in subsequent in vitro and in vivo experiments.

### 3.2. Liposome-Mediated Protection of Loaded pDNA

**Figure 2:**
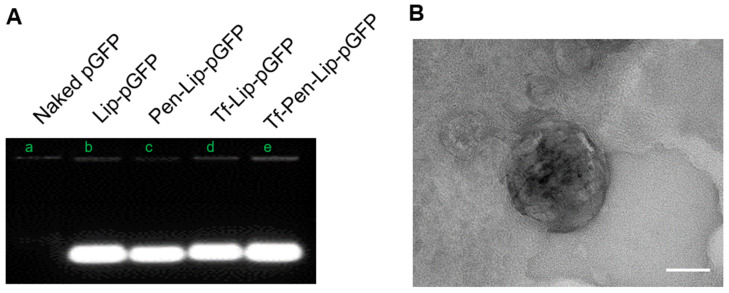
DNase protection assay and electron micrographs of liposomal formulations. (**A**). The representative image showing pGFP band after treatment with DNase I detected with electrophoresis on agarose gel. (**B**). Transmission electron microscopy (TEM) images showing the surface morphology of Tf-Pen-Liposome. Samples were stained with 1% phosphotungstic acid aqueous solution and observed using a JEM-2100 transmission electron microscope (Tokyo, Japan). The scale in the image is 50 nm.

To achieve successful gene delivery across the BBB into the brain, it is crucial for liposomes to protect pDNA from degradation by nucleases, which are present both inside and outside cells. The protective effect of liposomes was evaluated by incubation with DNase I followed by electrophoresis. As shown in Figure 2A, naked pGFP without liposomes was completely degraded by DNase, as indicated by the absence of the DNA band. In contrast, all liposome formulations effectively protected the encapsulated pGFP from DNase-mediated degradation. These results suggest that liposomes can serve as efficient vectors for transporting pDNA into cells without degradation. 

The morphological characteristics of the liposome were visualized using transmission electron microscopy (JEM-2100) after staining with 1% phosphotungstic acid aqueous solution. The electron micrograph is presented in Figure 2B, providing confirmatory evidence of the morphological attributes.

### 3.3. In Vitro Transfection Efficacy Assessment

**Figure 3:**
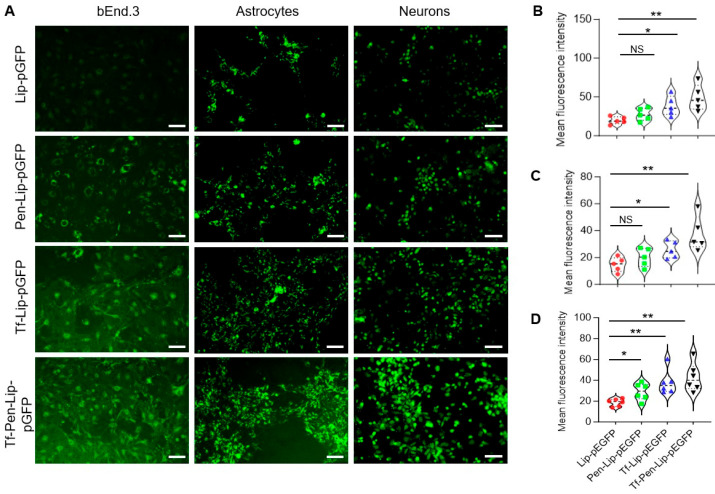
In vitro transfection efficacy of liposomes. (**A**). Representative fluorescence images of endothelial cells (bEnd.3, left), primary astrocytes (middle), and primary neurons (right) after treatment with Lip-pGFP, Pen-Lip-pGFP, Tf-Lip-pGFP, or Tf-Pen-Lip-pGFP. Scale bar = 75 μm. (**B**–**D**). Violin plots showing EGFP fluorescence intensity in bEnd.3 cells (**B**), primary astrocytes (**C**), and primary neurons (**D**) following liposome-mediated transfection. Data are presented as mean ± SEM (*n* = 5–6 independent experiments). Fluorescence intensity was quantified using ImageJ (version 1.54p) and normalized to image area. Statistical significance: NS, *p* > 0.05; * *p* < 0.05; ** *p* < 0.01.

To evaluate liposome-mediated gene transfection efficiency, bEnd.3 cells, primary astrocytes, and neurons were treated with various liposomal formulations containing chitosan-pGFP complexes for 4 h. EGFP expression in these cells was examined using an immunofluorescence microscope (Leica DMi8, Dear Park, IL, USA), and the fluorescence intensity was analyzed using ImageJ (version 1.54p). As shown in Figure 3, liposomes successfully mediated pGFP transduction in endothelial cells, astrocytes, and neurons. Surface modification with both Tf and Pen significantly enhanced pGFP transduction compared with liposomes modified with Tf or Pen alone. This enhanced transfection efficiency was observed across all tested cell types. The improved performance of dual-functionalized liposomes may be attributed to multiple factors, including increased cellular uptake, the protective effect of chitosan, PEGylation benefits, and the synergistic effect of the transferrin targeting ligand and cell-penetrating peptide. Collectively, these results demonstrate that dual-functionalized liposomes provide a robust platform for the efficient delivery of genetic material to brain cells.

### 3.4. In Vitro Detection of Transport Efficacy Across BBB Model

**Figure 4:**
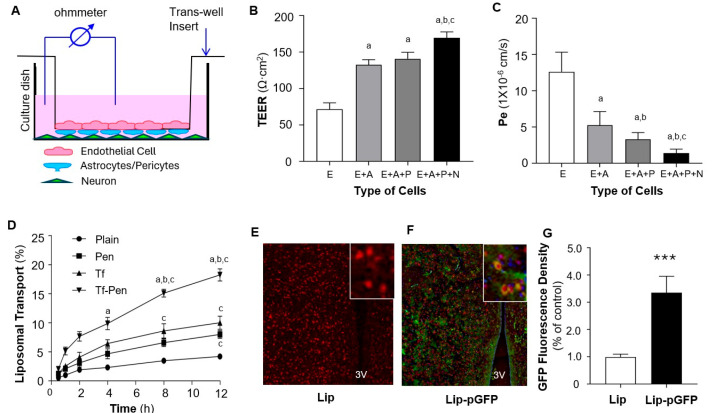
Transport efficiency of liposomes across the BBB in vitro and in vivo. (**A**). Schematic overview of the in vitro BBB model established using transwell co-cultures of endothelial cells (bEnd.3), astrocytes, pericytes, and neurons. (**B**). Bar graphs showing the functional integrity of BBB models established by different cell combinations: endothelial cells (E), endothelial cells plus astrocytes (E + A), endothelial cells plus astrocytes and pericytes (E + A + P), and endothelial cells plus astrocytes, pericytes, and neurons (E + A + P + N). BBB integrity was assessed by transepithelial electrical resistance (TEER). (**C**). Bar graphs showing the permeability of BBB models, measured as the permeability coefficient (Pe) of Na-F across the cell layers. Data are expressed as mean ± SEM (*n* = 4 independent experiments). Statistical significance: *p* < 0.05 compared with E alone (a), E + A (b), and E + A + P (c). (**D**). Transport of different liposomal formulations across the EAPN co-culture BBB model over 12 h. Transport efficiency was assessed using rhodamine-loaded liposomes: plain, Pen-, Tf-, and Tf-Pen–modified. Data are expressed as mean ± SEM (*n* = 4 independent experiments). Statistical significance: *p* < 0.05 compared with plain (a), Pen (b), and Tf (c). (**E**,**F**). Representative fluorescence images showing in vivo transport of Tf-Pen-liposomes across the BBB into the PVN of rat brains. Rats received intravenous injection of pGFP encapsulated in Tf-Pen-liposomes (**F**) or Tf-Pen-liposomes alone (**E**). EGFP expression in PVN neurons was detected by immunohistochemistry using anti-NeuN (neuronal marker, red) and anti-GFP (green). The third ventricle (3V) is labeled; insets show higher-magnification views. (**G**). Bar graphs showing GFP fluorescence intensity in the PVN region of rat brain sections. Data are expressed as mean ± SEM (*n* = 10 sections from 5 rats per group). *** *p* < 0.01 vs. control (Tf-Pen-liposomes alone).

To evaluate liposome-mediated transport across the BBB, we developed an in vitro BBB model consisting of co-cultures of endothelial cells, astrocytes, pericytes, and neurons, as shown in Figure 4A. The BBB integrity was assessed by measuring TEER across the cellular layers using electrophysiological methods. TEER was evaluated under four conditions: bEnd.3 monolayer, bEnd.3/astrocytes, bEnd.3/astrocytes/pericytes, and bEnd.3/astrocytes/pericytes/neurons. As shown in Figure 4B, the addition of astrocytes and pericytes markedly increased barrier integrity, and the inclusion of neurons further strengthened barrier function.

Next, we assessed the permeability of this BBB model by measuring the permeability coefficient (Pe) using sodium fluorescein (Na-F) as a marker due to its ability to diffuse across compromised barriers [32]. Na-F was added to the upper chamber of the inserts, and fluorescence intensity was measured to calculate Na-F concentrations and permeability coefficients, as described in our previous publication [24]. The results (Figure 4C) show that the Pe values strongly correlated with TEER measurements, indicating that the addition of astrocytes, pericytes, and neurons dramatically reduced barrier permeability. Collectively, these data demonstrate that the co-culture containing all four cell types (EAPN) exhibits high electrical resistance and low Na-F permeability, effectively mimicking the in vivo BBB. This model was thus used to evaluate our liposome-mediated delivery system.

The EAPN co-culture BBB model was then used to assess the transport efficiency of liposome-mediated BBB penetration. Transport efficacy was measured using rhodamine-encapsulated liposomes with various surface modifications, including plain liposomes (no conjugation), Pen, Tf, and both Tf-Pen. Fluorescence intensity of rhodamine was quantified to assess liposome transport. As shown in Figure 4D, liposome-mediated transport of rhodamine across the BBB model was time-dependent. Surface modification with Pen or Tf significantly enhanced liposome transport across the BBB. Notably, dual-functionalized liposomes conjugated with both Pen and Tf further promoted transport efficiency. Based on these results, Tf-Pen-liposomes (TP-Lip) were selected for subsequent in vivo gene delivery to the brain in rats.

### 3.5. In Vivo Transfection Efficacy Assay

The in vivo transport efficacy of Tf-Pen-liposomes across the BBB was evaluated in rats via intravenous injection of TP-Lip encapsulating pGFP (TP-pGFP). TP-Lip without pGFP was used as a control. GFP expression and localization in PVN neurons were detected in the brain sections using immunohistochemistry with antibodies against NeuN (a neuronal marker, red) and GFP (green). As shown in Figure 4E–G, IV injection of TP-pGFP dramatically increased GFP expression in the brain parenchyma, including neurons within the PVN. In contrast, GFP expression was not detectable in rats that received IV injection of TP-Lip alone. These results indicate that the dual-functionalized liposome (Tf-Pen-Lip) successfully delivered the *EGFP* gene across the BBB into brain cells and increased EGFP expression within the PVN, a major cardiovascular regulatory region, following systemic administration. Based on these findings, Tf-Pen-Lip was subsequently used for delivery of pACE2 across the BBB in further experiments.

### 3.6. Tf-Pen-Lip-pACE2 Attenuates Ang II-Induced Neurogenic Hypertension in Rats

**Figure 5:**
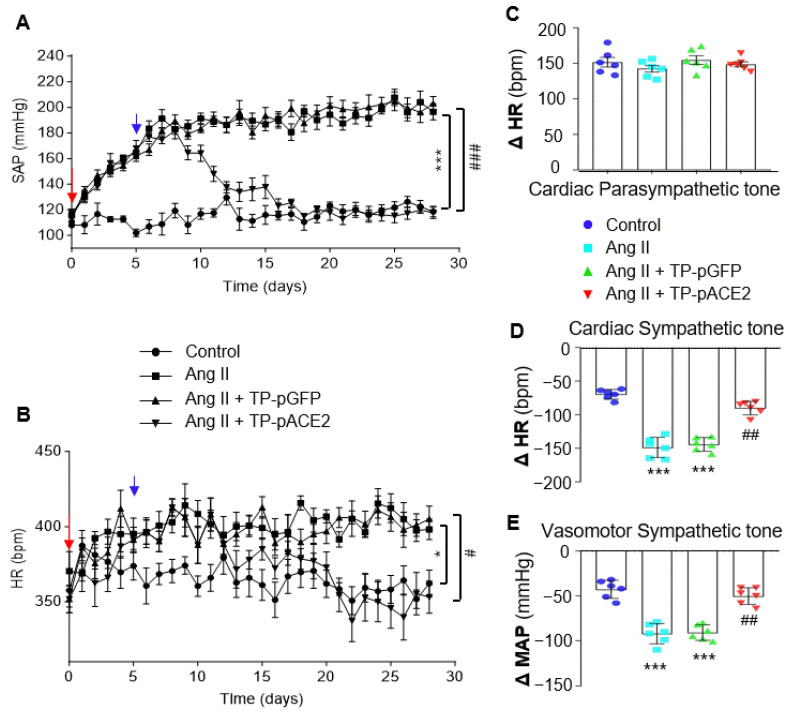
Effects of ICV infusion of Ang II and/or IV injection of Tf-Pen-Lip-pACE2 on BP, HR, and autonomic tone in rats. (**A**,**B**): Chronic BP (**A**) and HR (**B**) were measured in rats using the tail-cuff method before and after treatment with aCSF (control), Ang II, Ang II plus Tf-Pen-Lip-pGFP (TP-pGFP), or Ang II plus Tf-Pen-Lip-pACE2 (TP-pACE2). The red arrow indicates baseline BP and HR prior to treatment; the blue arrow indicates the day of IV injection with TP-pACE2 or TP-pGFP. Data are presented as mean ± SEM (*n* = 6 rats per group). Statistical significance was assessed by two-way ANOVA with Tukey’s multiple comparison test. * *p* <0.05, *** *p* < 0.001 vs. control; ^#^ *p* < 0.05, ^###^ *p* < 0.001 vs. TP-pGFP. (**C**): HR changes before and after intraperitoneal injection of atropine (1 mg/kg, muscarinic antagonist), reflecting cardiac parasympathetic tone. (**D**): HR changes before and after intraperitoneal injection of propranolol (4 mg/kg, β-blocker), reflecting cardiac sympathetic tone. (**E**): Mean arterial pressure (MAP) changes before and after administration of chlorisondamine (5 mg/kg, ganglionic blocker; nAChR antagonist), reflecting vasomotor sympathetic tone. Data are presented as mean ± SEM (*n* = 6 rats per group). Statistical significance was assessed using a two-tailed Student’s *t* test for two-group comparisons. *** *p* < 0.001 vs. aCSF control; ^##^ *p* < 0.01 vs. TP-pGFP.

To generate a neurogenic hypertension model, Ang II was infused into the brain via left cerebral lateral ventricular cannulation with osmotic minipump implantation in SD rats. BP and HR were recorded daily using a non-invasive tail-cuff system before and after treatment with aCSF (control), Ang II, Ang II plus Tf-Pen-Lip-pACE2, or Ang II plus Tf-Pen-Lip-pGFP. aCSF and pGFP served as controls. As shown in Figure 5A,B, chronic ICV infusion of Ang II (20 ng/min) significantly increased systolic BP, starting three days post-infusion, peaking within a week, and persisting for the subsequent three weeks. HR was also greatly elevated following Ang II infusion. In contrast, ICV infusion of aCSF did not alter BP or HR. Notably, systemic administration of Tf-Pen-Lip-pACE2 markedly attenuated Ang II–induced increases in BP and HR, with effects observed five days after IV injection and persisting throughout the recording period. Tf-Pen-Lip-pGFP did not alter Ang II–induced BP or HR changes.

To further examine the effects of Tf-Pen-Lip-pACE2 on neurogenic hypertension, autonomic activity was assessed in those four groups of rats. Cardiac parasympathetic tone, measured by HR response to atropine (a muscarinic antagonist), was not significantly altered by either Ang II or Tf-Pen-Lip-pACE2 (Figure 5C). Cardiac sympathetic tone, evaluated by HR response to propranolol (a β-adrenoreceptor blocker), was remarkably enhanced by Ang II infusion compared with aCSF. Systemic administration of Tf-Pen-Lip-pACE2 markedly attenuated Ang II–induced sympathetic activation by approximately 87%, whereas Tf-Pen-Lip-pGFP had no significant effect (Figure 5D). Vasomotor sympathetic activity, assessed by BP response to chlorisondamine (a ganglionic blocker), was similarly elevated by Ang II and effectively reduced by Tf-Pen-Lip-pACE2 treatment but not by Tf-Pen-Lip-pGFP (Figure 5E).

**Figure 6:**
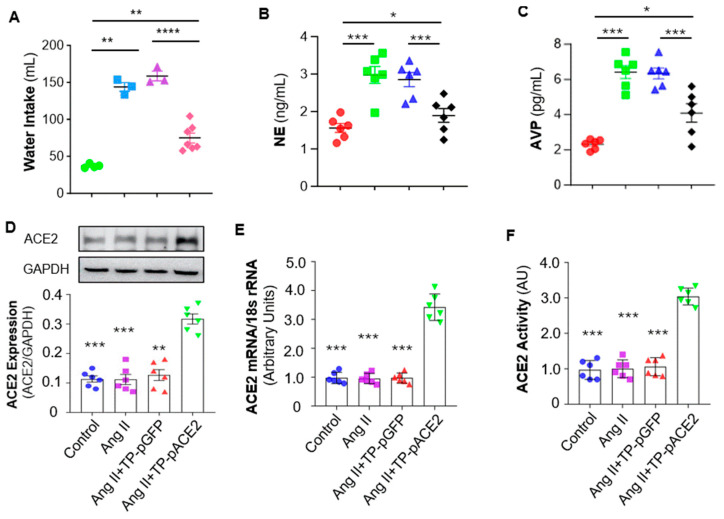
Effect of Ang II ICV infusion and Tf-Pen-Lip-pACE2 IV injection on water intake, plasma AVP and NE levels, and ACE2 expression in the PVN of rats. (**A**–**C**): The scatter dot plots showing the mean daily water intake (average in the final week of treatment) (**A**), plasma AVP (**B**) and NE (**C**) after treatment with aCSF, Ang II, Ang II plus Tf-Pen-Lip-pGFP (TP-pGFP), and Ang II plus Tf-Pen-Lip-pACE2 (TP-pACE2). Data are presented as mean ± SEM, *n* = 6 rats from each group. Statistical significance was assessed using 2-tailed Student’s *t* test for comparing 2 groups, * *p* < 0.05, ** *p* < 0.01, *** *p* < 0.001, **** *p* < 0.0001. (**D**–**F**): The bar graphs showing the ACE2 protein expression detected using Western Blots (**D**), ACE2 mRNA levels detected using real-time qPCR (**E**), ACE2 activity measured using ACE2 fluorogenic substrate (**F**). Data are presented as mean ± SEM, *n* = 3 independent experiments from 6 rats in each group. Statistical significance was assessed using 2-tailed Student’s *t* test for comparing 2 groups, ** *p* < 0.05, *** *p* < 0.001 as compared with Ang II + TP-pACE2.

It is well known that sympathetic nerve activity is regulated by parvocellular neurons in the PVN, which project to preganglionic neurons in the rostral ventrolateral medulla (RVLM) and intermediolateral cell column (IML), ultimately innervating target organs via norepinephrine (NE) release through postganglionic nerve terminals [33]. Therefore, plasma NE levels, a reliable indicator of sympathetic activation, were examined. The results (Figure 6B) indicated that plasma NE levels were markedly increased by Ang II ICV infusion compared with aCSF infusion, and that systemic administration of Tf-Pen-Lip-pACE2 markedly attenuated this rise, whereas Tf-Pen-Lip-pGFP had no significant effect.

In addition to parvocellular neurons, magnocellular neurons in the PVN project to the posterior pituitary to regulate arginine vasopressin (AVP) secretion, which reduces sodium and water excretion, up-regulating blood pressure [34]. Ang II also stimulates the thirst center via AT1 receptors, increasing water intake and blood volume [35]. Accordingly, in the current study, Ang II infusion significantly increased both water intake and plasma AVP levels compared with aCSF (Figure 6A,C). Systemic Tf-Pen-Lip-pACE2 attenuated these effects, whereas Tf-Pen-Lip-pGFP did not. Taken together, chronic ICV infusion of Ang II induces neurogenic hypertension characterized by elevated BP and HR, increased sympathetic activity, enhanced AVP secretion, and altered fluid homeostasis. Importantly, systemic administration of Tf-Pen-liposomes loaded with pACE2 substantially attenuated these Ang II–induced effects.

### 3.7. IV Injection of Tf-Pen-Lip-pACE2 Increased ACE2 Expression and Activity in the Brain

The hypothalamic PVN plays a critical role in the development of neurogenic hypertension by acting as a central control center for sympathetic nerve activity and regulating AVP secretion [34,35]. We therefore examined ACE2 expression in the PVN of those four groups of rats (control, Ang II, Ang II + Tf-Pen-Lip-pGFP, and Ang II + Tf-Pen-Lip-pACE2) using Western blotting for protein expression and real-time PCR for mRNA levels. As shown in Figure 6D (Western blot) and Figure 6E (real-time PCR), ICV infusion of Ang II or IV injection of Tf-Pen-Lip-pGFP did not alter ACE2 expression in the PVN. In contrast, systemic administration of Tf-Pen-Lip-pACE2 significantly increased ACE2 expression in this key brain region, which regulates sympathetic nerve activity and body fluid homeostasis.

To assess whether the liposome-mediated overexpression of ACE2 in the brain was functional, the enzymatic activity of ACE2 was measured in PVN tissue micropunched from those rats using the Palkovits technique, employing a quenched fluorescent ACE2 substrate. As presented in Figure 6F, chronic ICV Ang II infusion or IV Tf-Pen-Lip-pGFP injection did not affect ACE2 activity in the PVN. However, systemic administration of Tf-Pen-Lip-pACE2 markedly enhanced ACE2 enzymatic activity, demonstrating that the overexpressed ACE2 in the brain parenchyma is functionally active and likely contributes to the therapeutic effects on neurogenic hypertension observed in this study.

### 3.8. Biosafety Assessment of Tf-Pen-Lip-pACE2

**Figure 7:**
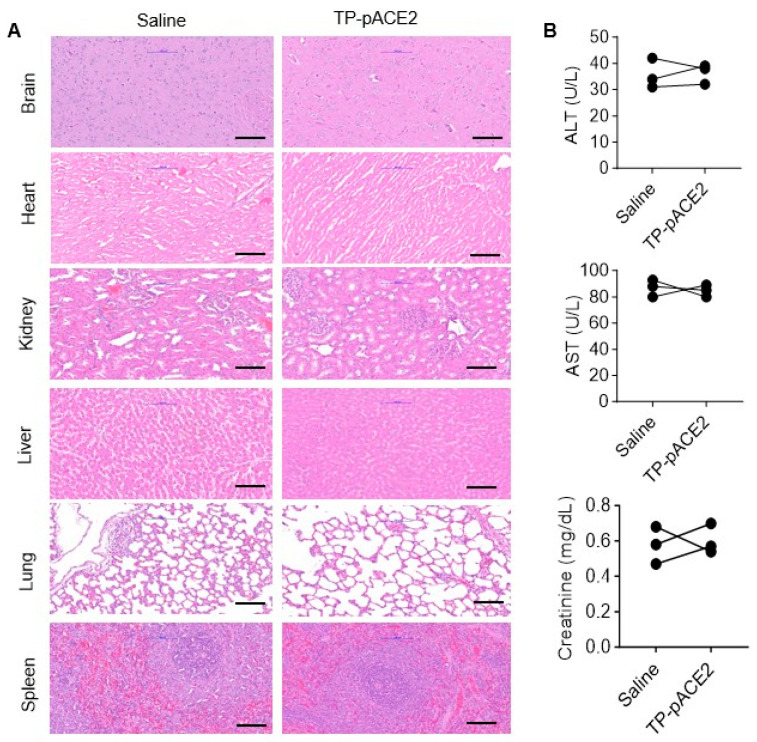
In vivo biocompatibility assessment of Tf-Pen-Lip-pACE2. To evaluate potential side effects of Tf-Pen-Lip-pACE2 (TP-pACE2), histological analysis of vital organs and serum biomarkers of tissue injury were examined in rats following one week of treatment with either saline (control) or TP-pACE2 (intravenous injection). (**A**). Representative H&E-stained sections of heart, liver, kidney, brain, lung, and spleen from each group (scale bar = 100 μm). (**B**). Blood was collected after treatment, and serum markers of organ damage were measured. Liver injury markers ALT (upper panel) and AST (middle panel), as well as the kidney injury marker creatinine (lower panel), are shown in (**B**) panel. Data are presented as mean ± SEM (*n* = 3). Statistical significance was assessed using a two-tailed Student’s *t* test for comparisons between two groups. Differences were not statistically significant (*p* > 0.05).

Biosafety is a key consideration for all therapeutic agents. To evaluate the biocompatibility of Tf-Pen-Lip-pACE2, SD rats were administered IV injections under the same conditions as in the previous experiments. Histological analysis of major organs was performed using H&E staining of tissue sections from rats treated with Tf-Pen-Lip-pACE2 or saline control. As shown in Figure 7A, no obvious tissue damage was observed in the Tf-Pen-Lip-pACE2–treated group compared with controls. Potential toxic effects on liver and kidney function were further assessed by measuring plasma levels of aspartate aminotransferase (AST), alanine aminotransferase (ALT), and creatinine. The results (Figure 7B) indicated no detectable impairment of liver or kidney function. Collectively, these findings suggest that systemic administration of Tf-Pen-Lip-pACE2 does not alter the morphology or function of major organs, demonstrating that this liposome-based delivery system possesses excellent biocompatibility.

## 4. Discussion

**Figure 8:**
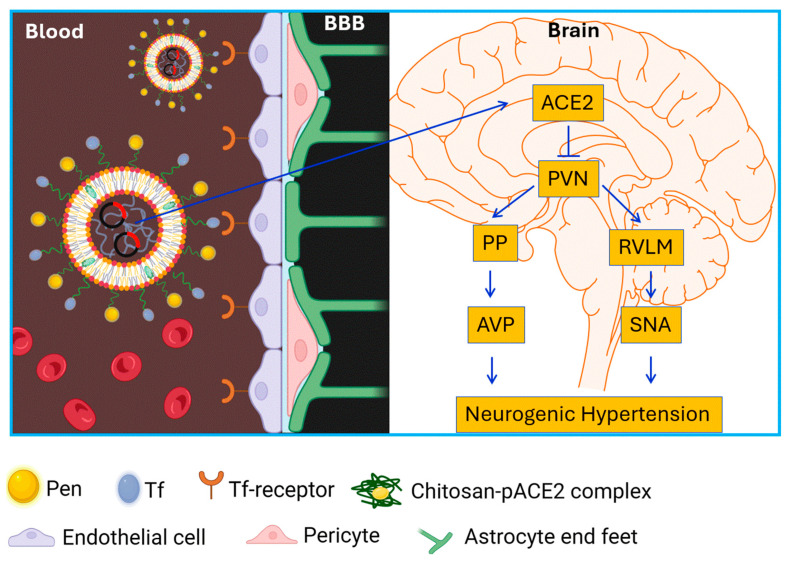
Schematic diagram showing liposome-mediated ACE2 gene delivery across the BBB on neurogenic hypertension. Pen: penetratin; Tf: transferrin; PVN: paraventricular nucleus; RVLM: rostral ventrolateral medulla; PP: post pituitary gland; AVP: arginine vasopressin; SNA: sympathetic nervous activity.

Hypertension affects nearly half of adults in the United States, approximately 120 million people [2]. Despite the availability of numerous antihypertensive drugs, the prevalence of uncontrolled or drug-resistant hypertension continues to rise, increasing the risk of end-organ damage, including stroke, chronic kidney disease, heart failure, and vascular pathologies, ultimately contributing to higher morbidity and mortality [3]. Recent clinical studies suggest that surgical restriction of sympathetic outflow can be effective for drug-resistant hypertension [4,5], highlighting a potential CNS origin—neurogenic hypertension. A hallmark of resistant hypertension is elevated sympathetic nerve activity. Early pharmacological approaches targeting sympathetic overactivity were often associated with significant adverse effects, and many other antihypertensive drugs are unable to efficiently cross the BBB due to physicochemical limitations [36]. To address these challenges, we designed and characterized a liposome-based gene delivery system capable of transporting the ACE2 gene across the BBB into key brain cardiovascular regulatory regions to control neurogenic hypertension (Figure 8).

PEGylated liposomes were selected for gene delivery due to several advantages: (1) biodegradability and lower immunogenicity compared with viral vectors, (2) protection of encapsulated DNA from nuclease degradation, ensuring prolonged stability and target accumulation, (3) capacity to carry large genetic fragments for long-lasting therapeutic effects, (4) amenability to surface modifications for cell- or tissue-specific targeting [37], and (5) demonstrated success in clinical applications, such as COVID-19 mRNA vaccines, mRNA-1273/SpikeVax developed by Moderna and BNT162b2/Comirnaty from BioNTech/Pfizer, as described in the Nobel Prize in Medicine 2023 (https://www.nobelprize.org/prizes/medicine/2023/advanced-information/) (accessed on 12 September 2025). To enhance BBB transport, liposomes were surface-modified with transferrin (Tf), a ligand facilitating receptor-mediated transcytosis, and penetratin (Pen), a cell-penetrating peptide that promotes cellular uptake. Chitosan was used to stabilize the pDNA complex, providing endosomal buffering, biodegradability, and low toxicity. The synthesized liposomes had a mean diameter of ~150 nm, a PDI below 0.3, and a slightly positive surface charge due to the cationic phospholipid DOTAP. Other major excipients in this liposomal formulation were carefully selected to ensure stability and functionality. Dioleoyl-3-trimethylammonium-propane chloride (DOTAP) was incorporated to impart a slight positive surface charge, thereby stabilizing the formulation and minimizing aggregation or phase separation. Dioleoyl-sn-glycerol-3-phosphoethanolamine (DOPE) was included as a helper lipid to enhance fusion with cellular and endosomal membranes while reducing the cytotoxicity typically associated with cationic lipids. Polyethylene glycol (PEG) was added to improve colloidal stability and limit clearance by the reticuloendothelial system, and cholesterol was incorporated to increase bilayer fluidity and stability. Importantly, our results indicate that this liposomal formulation provided effective protection of pDNA against nuclease-mediated degradation, underscoring its potential for BBB-targeted gene delivery. Collectively, this study provides the first evidence that these strategies remarkably enhance BBB penetration, gene delivery efficiency, and the biosafety of a brain-targeted delivery system. Using this dual-functionalized liposomal platform, we successfully achieved systemic delivery of the ACE2 gene across the BBB into the rat brain.

The transwell 2D BBB model, originally developed using a monolayer of cultured brain endothelial cells, has been widely applied to evaluate the passage of compounds and therapeutic agents across the BBB [38]. In this study, we refined the in vitro BBB model by incorporating additional brain cell types that reflect the anatomical complexity of the BBB, including astrocytes, pericytes, and neurons. Barrier evaluation studies demonstrated that the inclusion of astrocytes and pericytes markedly improved barrier integrity and reduced permeability. More importantly, co-culture with neurons further enhanced barrier function, rendering it more comparable to in vivo BBB properties. Although the underlying mechanisms remain incompletely understood, several recent studies provide plausible explanations: (1) neurons secrete factors such as glial cell line-derived neurotrophic factor, which upregulates tight junction protein expression in endothelial cells and reduces barrier permeability [39]; and (2) neurons establish direct contact and innervation with brain endothelial cells, creating signaling pathways that modulate BBB permeability [40]. Taken together, our results indicate that neurovascular unit formation in the EAPN BBB model contributes to the maintenance of BBB integrity and permeability. This model represents a valuable platform for evaluating agent transport across the BBB, studying BBB function under pathological conditions, and investigating neurovascular unit interactions in vitro. In the present study, the transport efficiency of plain liposomes (Lip), transferrin-conjugated liposomes (Tf-Lip), penetratin-conjugated liposomes (Pen-Lip), and dual-conjugated liposomes (Tf-Pen-Lip) was assessed using this improved BBB model. The results demonstrated that dual conjugation with both Tf and Pen markedly enhanced liposome-mediated transport across the BBB. Based on these findings, Tf-Pen-liposomes were selected for subsequent in vivo studies. Systemic administration of Tf-Pen-Lip-pGFP in rats resulted in robust EGFP expression in the hypothalamic PVN, confirming efficient BBB crossing and successful gene delivery into the brain.

To investigate therapeutic efficacy, a neurogenic hypertension model was established by chronic intracerebroventricular (ICV) infusion of Ang II in rats. This model recapitulates clinical neurogenic hypertension, characterized by elevated blood pressure (BP) and heart rate (HR), increased sympathetic tone, and enhanced arginine vasopressin (AVP) secretion. In Ang II–induced neurogenic hypertensive rats, systemic administration of Tf-Pen-Lip-pACE2 markedly increased ACE2 expression and activity in the brain and, most importantly, robustly attenuated Ang II–induced BP elevation, AVP hypersecretion, and sympathetic overactivity. The key question raised by these findings is why increased ACE2 expression in the brain attenuates Ang II–induced neurogenic hypertension. Several mechanisms may explain these effects based on previous studies: (i) Enzymatic modulation: ACE2 cleaves neuromodulators such as Ang II and apelin, dampening neuronal activity in both the parvocellular and magnocellular PVN compartments, thereby reducing sympathetic outflow and AVP secretion [10]. (ii) Neurotransmission: ACE2 enhances GABAergic signaling, inhibiting presympathetic PVN neurons that drive elevated BP and HR [41]. (iii) Cellular protection: ACE2 reduces oxidative stress and proinflammatory signaling, both of which contribute to neurogenic hypertension [10,42]. Nevertheless, the precise mechanisms by which ACE2 regulates neuronal activity and modulates neurogenic hypertension remain to be fully elucidated and require further investigation.

In summary, this study developed a dual-functionalized liposome system, combining Tf-mediated receptor targeting and Pen-mediated cell membrane penetration, to enhance BBB transport and gene delivery. Systemic Tf-Pen-Lip-pACE2 injection increased ACE2 expression in PVN neurons, attenuated Ang II–induced neurogenic hypertension, and normalized sympathetic and fluid balance. These findings demonstrate the potential of liposome-based gene therapy for treating neurogenic, drug-resistant hypertension. Future studies are warranted to evaluate long-term efficacy and safety, as well as applicability in additional hypertension models and human subjects. This research represents an important step toward developing brain-targeted therapeutic strategies for CNS–driven hypertension.

## Data Availability

Data will be available upon request.

## References

[1] Kario K., Okura A., Hoshide S., Mogi M. (2024). The WHO Global report 2023 on hypertension warning the emerging hypertension burden in globe and its treatment strategy. Hypertens. Res..

[2] Richardson L.C., Vaughan A.S., Wright J.S., Coronado F. (2024). Examining the hypertension control cascade in adults with uncontrolled hypertension in the US. JAMA Netw. Open.

[3] Carey R.M., Sakhuja S., Calhoun D.A., Whelton P.K., Muntner P. (2019). Prevalence of apparent treatment-resistant hypertension in the United States. Hypertension.

[4] Mufarrih S.H., Qureshi N.Q., Khan M.S., Kazimuddin M., Secemsky E., Bloch M.J., Giri J., Cohen D., Swaminathan R.V., Feldman D.N. (2024). Randomized trials of renal denervation for uncontrolled hypertension: An updated meta-analysis. J. Am. Heart Assoc..

[5] de Leeuw P.W., Bisognano J.D., Bakris G.L., Nadim M.K., Haller H., Kroon A.A. (2017). Sustained reduction of blood pressure with baroreceptor activation therapy: Results of the 6-year open follow-up. Hypertension.

[6] Scheffers I.J., Kroon A.A., Schmidli J., Jordan J., Tordoir J.J., Mohaupt M.G., Luft F.C., Haller H., Menne J., Engeli S. (2010). Novel baroreflex activation therapy in resistant hypertension: Results of a European multi-center feasibility study. J. Am. Coll. Cardiol..

[7] Rippy M.K., Zarins D., Barman N.C., Wu A., Duncan K.L., Zarins C.K., Rippy M.K. (2011). Catheter-based renal sympathetic denervation: Chronic preclinical evidence for renal artery safety. Clin. Res. Cardiol..

[8] Dampney R.A., Michelini L.C., Li D.P., Pan H.L. (2018). Regulation of sympathetic vasomotor activity by the hypothalamic paraventricular nucleus in normotensive and hypertensive states. Am. J. Physiol. Heart Circ. Physiol..

[9] Iovino M., Lisco G., Giagulli V.A., Vanacore A., Pesce A., Guastamacchia E., De Pergola G., Triggiani V. (2021). Angiotensin II-vasopressin interactions in the regulation of cardiovascular functions. Evidence for an impaired hormonal sympathetic reflex in hypertension and congestive heart failure. Endocr. Metab. Immune Disord. Drug Targets.

[10] Mohammed M., Berdasco C., Lazartigues E. (2020). Brain angiotensin converting enzyme-2 in central cardiovascular regulation. Clin. Sci..

[11] Santos R.A.S., Sampaio W.O., Alzamora A.C., Motta-Santos D., Alenina N., Bader M., Campagnole-Santos M.J. (2018). The ACE2/Angiotensin-(1-7)/MAS axis of the renin-angiotensin system: Focus on angiotensin-(1-7). Physiol. Rev..

[12] Feng Y., Xia H., Cai Y., Halabi C.M., Becker L.K., Santos R.A., Speth R.C., Sigmund C.D., Lazartigues E. (2010). Brain-selective overexpression of human Angiotensin-converting enzyme type 2 attenuates neurogenic hypertension. Circ. Res..

[13] Yamazato M., Yamazato Y., Sun C., Diez-Freire C., Raizada M.K. (2007). Overexpression of angiotensin-converting enzyme 2 in the rostral ventrolateral medulla causes long-term decrease in blood pressure in the spontaneously hypertensive rats. Hypertension.

[14] Sehlin D., Hultqvist G., Michno W., Aguilar X., Dahlén A.D., Cerilli E., Bucher N.M., Lopes S., Syvänen S. (2025). Bispecific brain-penetrant antibodies for treatment of Alzheimer’s disease. J. Prev. Alzheimer’s Dis..

[15] Okuyama T., Eto Y., Sakai N., Minami K., Yamamoto T., Sonoda H., Yamaoka M., Tachibana K., Hirato T., Sato Y. (2019). Iduronate-2-sulfatase with anti-human transferrin receptor antibody for neuropathic mucopolysaccharidosis II: A phase 1/2 trial. Mol. Ther..

[16] Bell R.D., Ehlers M.D. (2014). Breaching the blood-brain barrier for drug delivery. Neuron.

[17] Niewoehner J., Bohrmann B., Collin L., Urich E., Sade H., Maier P., Rueger P., Stracke J.O., Lau W., Tissot A.C. (2014). Increased brain penetration and potency of a therapeutic antibody using a monovalent molecular shuttle. Neuron.

[18] Liu C., Tai L., Zhang W., Wei G., Pan W., Lu W. (2014). Penetratin, a potentially powerful absorption enhancer for noninvasive intraocular drug delivery. Mol. Pharm..

[19] Modgil A., Zhang Q., Pingili A., Singh N., Yao F., Ge J., Guo L., Xuan C., O’Rourke S.T., Sun C. (2012). Angiotensin-(1-7) attenuates the chronotropic response to angiotensin II via stimulation of PTEN in the spontaneously hypertensive rat neurons. Am. J. Physiol. Heart Circ. Physiol..

[20] Schildge S., Bohrer C., Beck K., Schachtrup C. (2013). Isolation and culture of mouse cortical astrocytes. J. Vis. Exp..

[21] Boroujerdi A., Tigges U., Welser-Alves J.V., Milner R. (2014). Isolation and culture of primary pericytes from mouse brain. Methods Mol. Biol..

[22] Lakkadwala S., Dos Santos Rodrigues B., Sun C., Singh J. (2019). Dual functionalized liposomes for efficient co-delivery of anti-cancer chemotherapeutics for the treatment of glioblastoma. J. Control. Release.

[23] Sharma G., Modgil A., Layek B., Arora K., Sun C., Law B., Singh J. (2013). Cell penetrating peptide tethered bi-ligand liposomes for delivery to brain in vivo: Biodistribution and transfection. J. Control. Release.

[24] Sharma G., Modgil A., Sun C., Singh J. (2012). Grafting of cell-penetrating peptide to receptor-targeted liposomes improves their transfection efficiency and transport across blood-brain barrier model. J. Pharm. Sci..

[25] Zhang Q., Yao F., Raizada M.K., O’Rourke S.T., Sun C. (2009). Apelin gene transfer into the rostral ventrolateral medulla induces chronic blood pressure elevation in normotensive rats. Circ. Res..

[26] Zhang Q., Yao F., O’Rourke S.T., Qian S.Y., Sun C. (2009). Angiotensin II enhances GABA(B) receptor-mediated responses and expression in nucleus tractus solitarii of rats. Am. J. Physiol. Heart Circ. Physiol..

[27] Yao F., Sumners C., O’Rourke S.T., Sun C. (2008). Angiotensin II increases GABAB receptor expression in nucleus tractus solitarii of rats. Am. J. Physiol. Heart Circ. Physiol..

[28] Sriramula S., Pedersen K.B., Xia H., Lazartigues E. (2017). Determining the enzymatic activity of angiotensin-converting enzyme 2 (ACE2) in brain tissue and cerebrospinal fluid using a quenched fluorescent substrate. Methods Mol. Biol..

[29] Petros R.A., DeSimone J.M. (2010). Strategies in the design of nanoparticles for therapeutic applications. Nat. Rev. Drug Discov..

[30] Saraiva C., Praça C., Ferreira R., Santos T., Ferreira L., Bernardino L. (2016). Nanoparticle-mediated brain drug delivery: Overcoming blood–brain barrier to treat neurodegenerative diseases. J. Control. Release.

[31] Holsæter A.M., Wizgird K., Karlsen I., Hemmingsen J.F., Brandl M., Škalko-Basnet N. (2022). How docetaxel entrapment, vesicle size, zeta potential and stability change with liposome composition-A formulation screening study. Eur. J. Pharm. Sci..

[32] Ahishali B., Kaya M. (2021). Evaluation of blood-brain barrier integrity using vascular permeability markers: Evans blue, sodium fluorescein, albumin-alexa fluor conjugates, and horseradish peroxidase. Methods Mol. Biol..

[33] Zhou J.J., Shao J.Y., Chen S.R., Ye Z.Y., Pan H.L. (2025). RCAN1-mediated calcineurin impairment drives sympathetic outflow in hypertension. Circ. Res..

[34] Huber M.J., Fan Y., Jiang E., Zhu F., Larson R.A., Yan J., Li N., Chen Q.H., Shan Z. (2017). Increased activity of the orexin system in the paraventricular nucleus contributes to salt-sensitive hypertension. Am. J. Physiol. Heart Circ. Physiol..

[35] Sandgren J.A., Linggonegoro D.W., Zhang S.Y., Sapouckey S.A., Claflin K.E., Pearson N.A., Leidinger M.R., Pierce G.L., Santillan M.K., Gibson-Corley K.N. (2018). Angiotensin AT_1A_ receptors expressed in vasopressin-producing cells of the supraoptic nucleus contribute to osmotic control of vasopressin. Am. J. Physiol. Regul. Integr. Comp. Physiol..

[36] Grassi G. (2023). Sympathetic modulation as a goal of antihypertensive treatment: From drugs to devices. J. Hypertens..

[37] Butt M.H., Zaman M., Ahmad A., Khan R., Mallhi T.H., Hasan M.M., Khan Y.H., Hafeez S., Massoud E.E.S., Rahman M.H. (2022). Appraisal for the potential of viral and nonviral vectors in gene therapy: A review. Genes.

[38] Rice O., Surian A., Chen Y. (2022). Modeling the blood-brain barrier for treatment of central nervous system (CNS) diseases. J. Tissue Eng..

[39] Yang L., Lin Z., Mu R., Wu W., Zhi H., Liu X., Yang H., Liu L. (2024). Neurons enhance blood-brain barrier function via upregulating claudin-5 and VE-cadherin expression due to glial cell line-derived neurotrophic factor secretion. Elife.

[40] Pulido R.S., Munji R.N., Chan T.C., Quirk C.R., Weiner G.A., Weger B.D., Rossi M.J., Elmsaouri S., Malfavon M., Deng A. (2020). Neuronal activity regulates blood-brain barrier efflux transport through endothelial circadian genes. Neuron.

[41] Mukerjee S., Gao H., Xu J., Sato R., Zsombok A., Lazartigues E. (2019). ACE2 and ADAM17 interaction regulates the activity of presympathetic neurons. Hypertension.

[42] Xia H., Suda S., Bindom S., Feng Y., Gurley S.B., Seth D., Navar L.G., Lazartigues E. (2011). ACE2-mediated reduction of oxidative stress in the central nervous system is associated with improvement of autonomic function. PLoS ONE.

